# Continuous Distillation for Drug Substance Manufacturing

**DOI:** 10.1007/s11095-026-04172-7

**Published:** 2026-08-06

**Authors:** Kimberly E. Penzer, Daniel G. Gregory, Justin T. Turnage, Colin D. Thieken, Samantha G. Kohn, James K. Ferri

**Affiliations:** Department of Chemical and Life Science Engineering, Virginia Commonwealth University, 601 West Main St., Richmond, VA 23284 USA

**Keywords:** advanced manufacturing technology, continuous in-line distillation, impurity removal, quality, separations

## Abstract

**Purpose:**

Continuous distillation is widely used in the commodity and petrochemical industry to purify fine chemicals on massive industrial scales; however, this key separation strategy is largely unused within the pharmaceutical industry. This article investigates the development of a pilot-scale continuous distillation system for in-line removal of an excess amine reactant (*i.e.*, tert-butylamine) when incorporated within an advanced manufacturing technology (AMT) for continuous drug substance production.

**Methods:**

Initial design parameters were simulated in ASPEN + to assess potential process solvents, setpoint parameters, and azeotrope formation. Complementary analytical characterization was performed with high performance liquid chromatography (HPLC) and gas chromatography – flame ionization detection (GC-FID). Early continuous distillation prototypes and key engineering design decisions are highlighted.

**Results:**

The final engineered system is comprised of a + 3-foot distillation column fitted with an Allihn condenser, a 250 mL three-necked round bottom flask, and an automated system for reflux ratio (R_D_) control. The column is readily integrated within an advanced manufacturing system to continuously remove amine reagent during continuous manufacturing of albuterol sulfate at a 3.0 mL/min process basis, corresponding to a theoretical throughput of approximately 1,800 doses per hour based on the demonstrated process flowrate, reaction stoichiometry, and operating conditions.

**Conclusions:**

The application of continuous distillation offers a unique strategy for impurity removal during advanced pharmaceutical manufacturing and can be scaled to meet target throughputs. Here we provide general criteria necessary for successful implementation of similar continuous in-line distillation systems.

**Graphical Abstract:**

**Figure:**
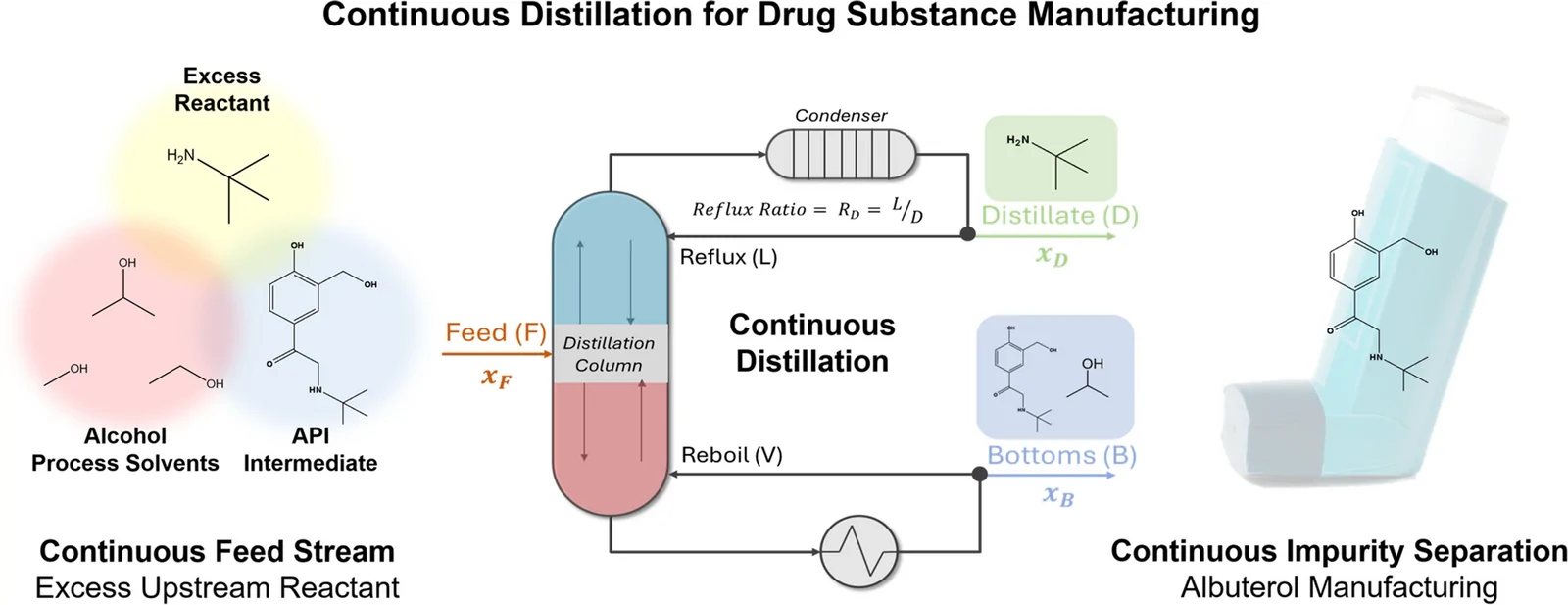

**Supplementary Information:**

The online version contains supplementary material available at https://doi.org/10.1007/s11095-026-04172-7.

## Introduction

### Continuous Distillation for In-Line Drug Substance Manufacturing – An Emerging Technology

Distillation has historically been utilized in the petrochemical industry to separate and purify molecular components on enormous production scales. Since classical antiquity, distillation has also found smaller-scale applications in the batch production of spirits and alcohol, with processing volumes ranging from approximately 10 to 10,000 gallons depending on the size of the distillery [1, 2]. Today, over 40,000 industrial distillation columns are currently in operation for the separation of nearly every commodity chemical transacting across the global marketplace [3]. Major distillation products include crude oil, solvents, and fine chemicals. A single crude oil refinery alone can process 90–100 million barrels of oil per year, which positions oil refining as the largest application of distillation in industry [4, 5]. Similarly, commodity chemicals are processed via continuous distillation to purify over + 100 million metric tons per year (e.g., methanol production) [6]. Thus, on a per mass basis, distillation is the largest separation technique employed by the chemicals industry.

**Fig. 1:**
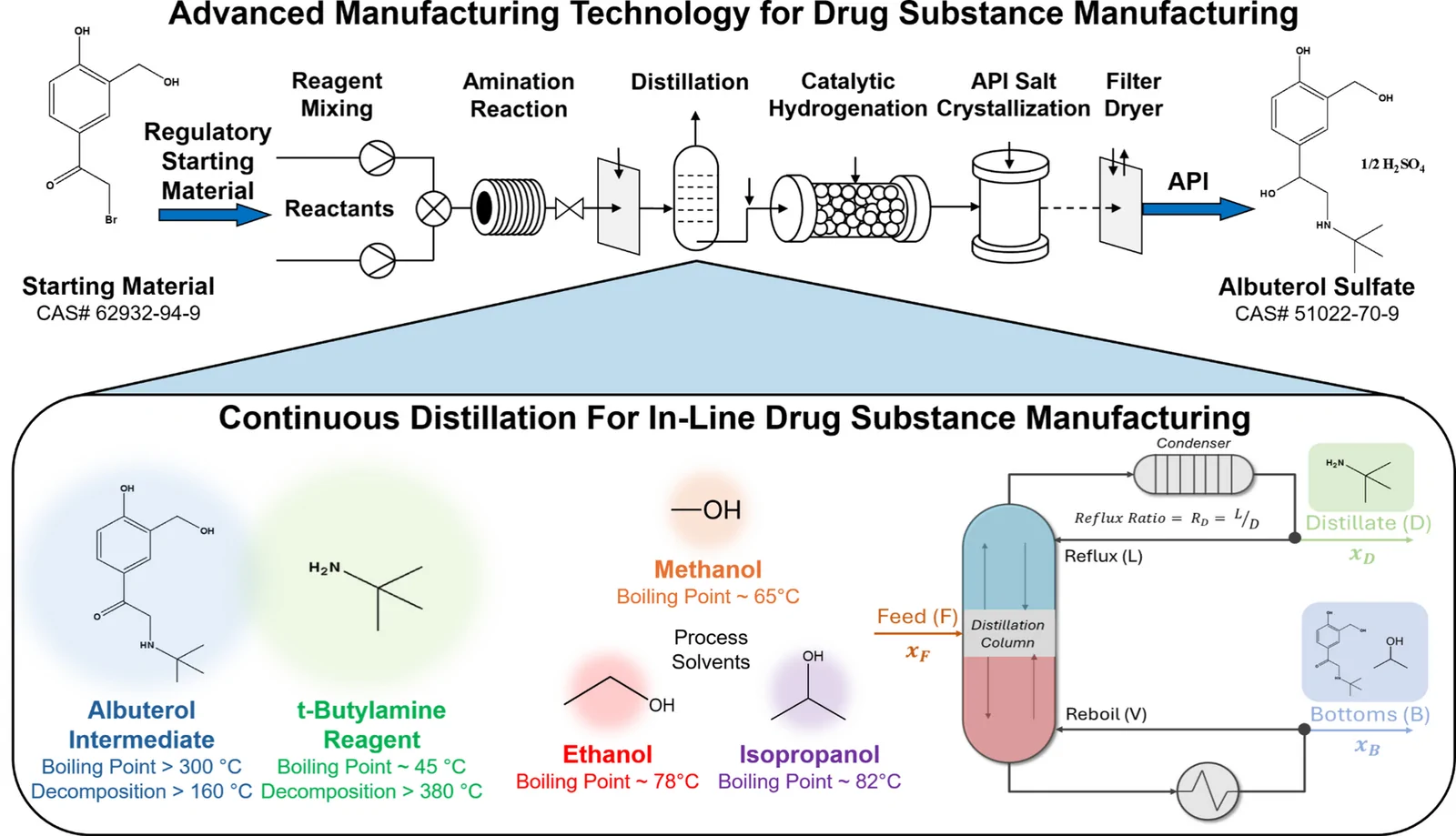
Integration of a continuous in-line distillation system within an advanced manufacturing technology for drug continuous substance production. Here an excess amine reactant (*i.e.*, tert-butylamine) is removed from the process stream via continuous in-line distillation. Aspects of this figure were modified and adapted in part with permission from ref. 13.. Copyright 2026 Daniel G. Gregory. Licensed under CC-BY 4.0.

However, while distillation is a key unit operation in the commodity chemicals industry, it is virtually unutilized for the production of active pharmaceutical ingredients (API’s). This is partly due to the comparatively small scales employed (*i.e.,* ≤ 1,000 metric tons per year) and the batch process strategies used for API manufacturing [7, 8]. Additionally, the high degree of pharmaceutical process variability has hindered the adoption of distillation strategies. Traditional batch separation techniques utilized for drug substance (DS) manufacturing include extraction, filtration, and recrystallization. However, the recent advent of continuous manufacturing (CM) [9] for drug substance production offers a unique opportunity for the integration of small-scale continuous distillation columns within automated API manufacturing systems [10]. In CM processes, pharmaceuticals are produced in a fashion analogous to a small-scale petrochemical refinery, with interlinked unit operations consisting of tubular plug flow reactors (PFR’s) [11], catalytic packed bed reactors (PBR’s) [12], continuously stirred tank reactors (CSTR’s) [13], and in-line filtration systems [14]. Thus, it is only natural to consider the incorporation of continuous in-line distillation systems in these emerging “*mini-pharmaceutical refineries*” (See Fig. 1).

The U.S. Food and Drug Administration (FDA) recently published their guidance for advanced manufacturing technologies (AMT’s) as a way of catalyzing the development of automated platforms for DS manufacturing [15]. This guidance sets the groundwork for advanced API manufacturing to relieve supply chain issues for lifesaving drugs, a global phenomenon which was recently exacerbated by the SARS CoV-2 virus [16–18]. In this latest FDA designation, an AMT is defined as a system which reduces development time of a drug or increases the supply of a substance on the FDA’s drug shortage list [19]. However, these emerging manufacturing platforms need a diverse toolbox of in-line purification strategies to enable continuous DS manufacturing to meet FDA guidelines. While optimization of reaction conditions can reduce excess reagent requirements and thereby lessen downstream separation demands, many pharmaceutical syntheses continue to employ excess reactants to maximize conversion, yield, or selectivity. Consequently, robust in-line purification strategies remain essential for continuous manufacturing platforms. Here we detail the integration of a continuous in-line distillation column within a candidate AMT for impurity removal during the production of albuterol sulfate, a bronchodilator asthma drug in chronic shortage (See Fig. 1). We then detail the strategy and general criteria necessary for integration of in-line distillation columns within continuous pharmaceutical manufacturing systems.

We previously detailed the construction of a continuous manufacturing system for albuterol sulfate production in a recent publication (See Supplementary Information (SI), Figures S1−2) [13]. The process incorporates (I) a laminar flow reactor (LFR) to enable an S_N_2 amination reaction, (II) a distillation column to remove excess reactant (*i.e.*, tert-butylamine, TBA), (III) a catalytic hydrogenation reactor, (IV) an API salt crystallization tank, and (V) an automated filter dryer to remove remaining impurities (e.g., dimerized salt compounds) [13]. Here we highlight the design and development of the continuous in-line distillation system (See Fig. 1) for the removal of an excess TBA during the manufacture of albuterol sulfate within short-chain alcohol process solvents (e.g., methanol, ethanol, and isopropanol). This system is currently under construction within a contract manufacturing organization (CMO) for DS production under current good manufacturing practices (cGMP). We plan to seek FDA designation of this emerging technology as an “AMT” for the production of albuterol sulfate.

### Strategy: Engineering a Continuous In-Line Distillation Column for API Manufacturing

**Fig. 2:**
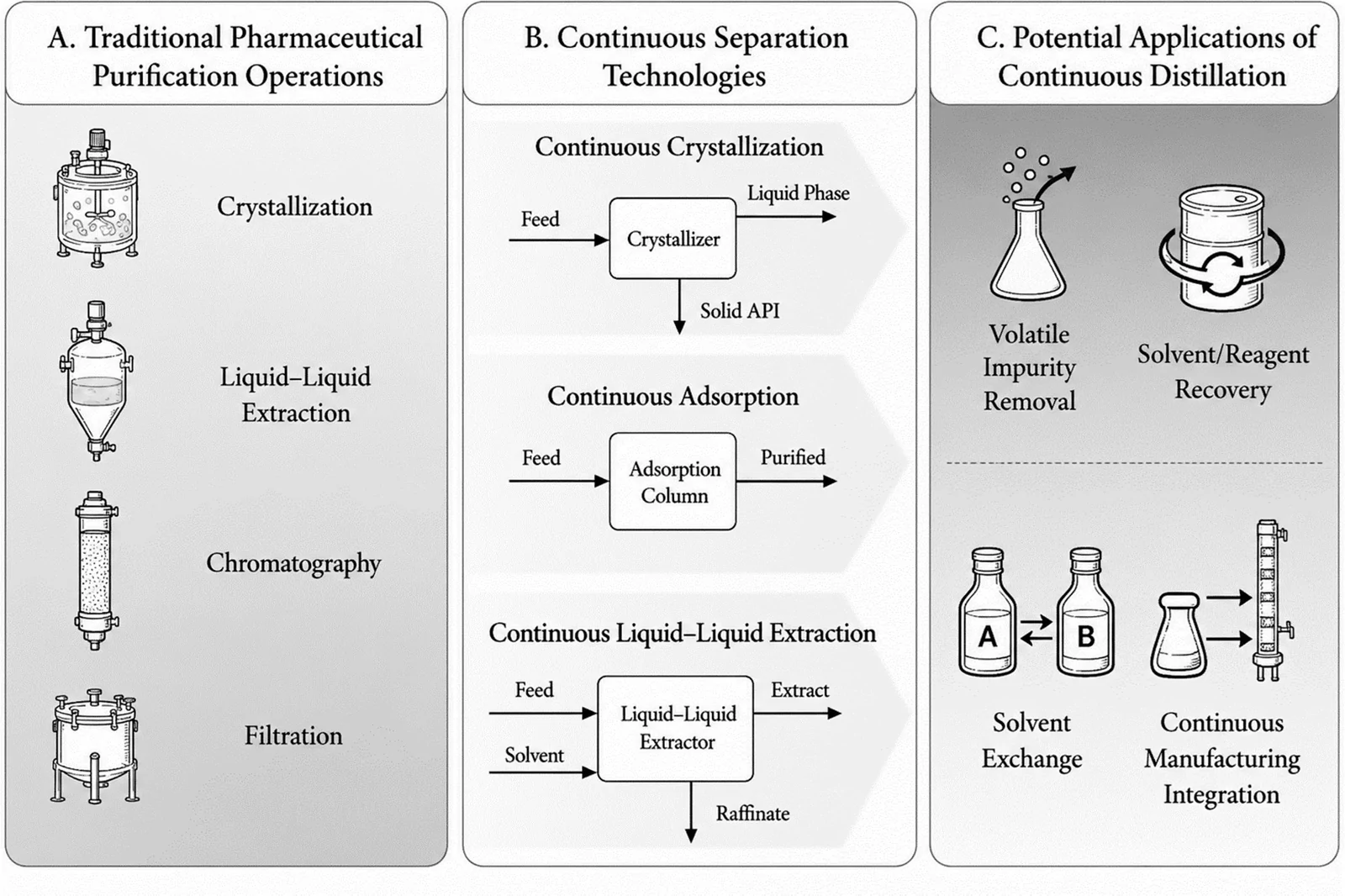
Overview of pharmaceutical purification and separation strategies. (**A**) Traditional pharmaceutical purification operations commonly employed during batch manufacturing, including crystallization, liquid–liquid extraction, chromatography, and filtration. (**B**) Representative continuous separation technologies including continuous crystallization, adsorption, and extraction. (**C**) Potential applications of continuous distillation within pharmaceutical manufacturing, including volatile impurity removal, solvent and reagent recovery, solvent exchange, and process integration.

Traditional batch pharmaceutical chemistry often employs S_N_2 amination reactions to functionalize the skeletal backbone of API intermediates with nitrogen-based pharmacophores (e.g. amines and amides). These reactions are often performed with excess amine equivalents (e.g. ~ 2–4 molar equiv.) in order to drive the reaction to completion [11]. However, the same reagent starting materials (SM’s) which drive the reaction to completion, must ultimately be removed via purification in order to satisfy the U.S. Food and Drug Administration’s guidelines for drug substance purity [20]. Fortunately, by heating a multi-component intermediate solution, one is able to distill or remove the more volatile components (*i.e.*, the lower boiling point SM’s) while simultaneously concentrating the heavier molecules (*i.e.*, higher boiling point intermediates). This process contrasts with traditional pharmaceutical purification operations, including crystallization, liquid–liquid extraction, chromatography, and filtration. Figure 2 highlights the toolbox of emerging continuous separation technologies and potential applications of continuous distillation within pharmaceutical manufacturing. Traditional pharmaceutical purification operations commonly employed in pharmaceutical batch manufacturing are summarized in Fig. 2A, while representative continuous separation technologies and potential applications of continuous distillation are summarized in Figs. 2B and C, respectively.

Each separation strategy offers unique advantages and disadvantages while their application depends on the overall process chemistry. Among the continuous separation technologies shown in Fig. 2B, distillation remains relatively underutilized within pharmaceutical manufacturing despite its potential applications in impurity removal, solvent recovery, and process integration (Fig. 2C). Lightweight amine reactants are often highly volatile and can be readily removed from higher boiling point solvents via heating, rotary evaporation, thin film evaporation, and distillation. Evaporative separation techniques are beneficial in that they limit the amount of solvents employed in the purification process, at the cost of added energy. Additionally, evaporative techniques can be applied to both solids and liquids. This helps to prevent yield losses commonly associated with washing and reduces the amount of waste generated.

Distillation can be accomplished either as a batch process or via in-line continuous distillation which incorporates an inlet feed stream along with an outlet distillate stream (*i.e.*, top stream) and a lower bottoms stream. Continuous distillation is scalable and can be tailored to meet the flowrate of a manufacturing process by increasing the column’s height, diameter, and internal packing. Additionally, in select applications, distillation offers the option to recycle recovered chemicals. The incorporation of in-line distillation has several advantages towards continuous DS manufacturing which have not been fully detailed in pharmaceutical literature. These benefits include (I) removal of solvents and excess starting materials, (II) removal of impurities, (III) prevention of downstream catalyst deactivation, (IV) streamlining downstream API salt formation processes, and (V) improving overall process efficiency.

Industrial distillation columns typically feature a tall central column ranging from ~ 1–100 m. The column’s internal architecture is selectively engineered to incorporate high surface area components (*i.e.*, trays or packing material) which provide a large interface between each phase. The fundamental purpose of the column’s internal structure is to provide ample surface area to enable molecular transport between the vapor and liquid phases. As the process stream is distilled, a thin liquid film coats the interior surfaces of the column to maintain vapor–liquid equilibrium (VLE) along each section of the column. This high surface area film provides ample interfacial surface area to enable mass transport of the molecular components across the VLE phase interface. The lower portion of the column (*i.e.*, the stripping portion) effectively heats the lightweight components for removal, whereas the upper portion of the column (*i.e.*, the rectifying portion) condenses the vapors to purify the lighter weight components (See SI Fig. S3). The exterior is thermally insulated to enable robust temperature control along the height of the column. Finally, automated distillate reflux control systems are included to rectify the evolving vapor at the top of the column, whereas a reboiling system is stationed at the base to heat the liquid. Key engineering design decisions include (I) the selection of the packing material *versus* tray architecture, (II) design of the height and width of the column (*i.e.*, staging), (III) selection of flowrates, (IV) reflux ratio (R_D_) optimization, and (V) plumbing the location of the feed inlet (*i.e.*, near the top, middle, or bottom of the column).

Engineering a large-scale distillation column is a complex capital-intensive process that traditionally involves an initial series of binary VLE experiments to determine the mole fraction of each component in both the vapor (y) and liquid phases (x). Next, engineers typically employ the McCabe–Thiele method to graphically scale the dimensions of a distillation column [21, 22]. In order to expedite the process, engineers have long sought the development of accurate equations of state (EOS) which would streamline the calculation of multi-component mixtures to enable faster scale-up without the need for extensive laboratory experiments. Multiple EOS and activity coefficient (γ) models have been developed including the Peng-Robinson equation, the Redlich-Kwong equation, the Universal Quasichemical Activity Coefficient model (UNIQUAC), the relative volatility model, and the Non-Random Two-Liquid model (NRTL) [23–27]. Equations of state are then utilized in process simulation software (e.g., ASPEN +) to model, scale-up, and optimize the construction of a distillation column. However, EOS models notoriously struggle to accurately model VLE curves for complex solutions as they struggle to account for the dynamic interactions between functional groups in multicomponent mixtures. Thus, researchers often utilize a combination of experiments, databases, and models to curate VLE diagrams [28, 29]. More recently however, neural networks (e.g., EquiNet) might provide a new avenue for rapid VLE predictions for complex chemical mixtures [30].

In the following sections we highlight our recent research and development of an in-line continuous distillation column for integration within a pilot plant for API purification. Initial simulations were performed in ASPEN + to obtain VLE curves to assess potential process solvents for TBA separation. Next, flash distillation experiments were conducted with binary mixtures of TBA in common alcohol solvents including methanol (MeOH), ethanol (EtOH), and isopropyl alcohol (IPA). A series of distillation prototypes were constructed including the development of laboratory benchtop distillation systems, a 3-foot prototype system, and the configuration of a custom blown glass distillation column for integration within a pilot plant. The construction of the continuous distillation column is detailed, and key engineering design decisions are highlighted including reflux automation and the incorporation of in-house process controls. The resulting system is capable of removing 2.0 molar equivalents of TBA for each API molecule when operated at a 3 mL/min basis. Lastly, a discussion is provided to assess the potential for incorporation of in-line distillation to meet FDA specifications for drug substance purity.

## Materials and Methods

### Synthesis of Aminated Process Material for Distillation

Stock aminated process material was prepared in flow for continuous distillation as reported elsewhere [11]. To briefly summarize, stock intermediate solutions were made by preparing a 100 mg/mL solution of 2-Bromo-1-[4-hydroxy-3-(hydroxymethyl)phenyl]ethan-1-one (*i.e.*, the SM, CAS# 62932–94-9) in anhydrous alcohol solvents (*i.e.*, MeOH, EtOH, or IPA). Next, a batch of t-butylamine (*i.e.*, TBA, CAS# 75–64-9) was prepared in a separate flask and diluted in alcohol to 120 mg/mL. The solutions were pumped to a Vapourtec E-Series flow reactor and mixed along a T-junction just before entering the reactor. The reactor was comprised of 1/8″ PTFE tubing which was coiled within a chamber and heated to 60°C. The reactants were pumped through the LFR under previously reported operating conditions. Depending on the selected reactor configuration and flowrate, residence times ranging from approximately 40–60 min were employed. Typical process flowrates ranged from 1–3 mL/min. The product stream was assessed with HPLC and GC-FID as discussed below. The resulting product consisted of 2-(tert-butylamino)−1-[4-hydroxy-3-(hydroxymethyl)phenyl]ethanone at ~ 40 mg/mL and was diluted as needed for continuous distillation.

### Binary Flash Distillation

Binary mixtures were prepared for flash distillation studies (*i.e.*, TBA-MeOH, TBA-EtOH, and TBA IPA) to develop experimental VLE curves for comparison *versus* models as simulated in ASPEN +. The binary flash column consisted of a Hemple distilling column (24/40 joint), a 250 mL three necked round bottom flask (RBF), a heating mantle, and a Dean-Stark tube. Binary solutions were prepared by dissolving TBA in each process solvent at mole fractions ranging from 0.01–0.12. The samples were then introduced to a binary flash column for the VLE studies. The system was heated to reflux, while samples were collected in triplicate for GC analysis at each mole fraction (*i.e.*, 0.01—0.12).

### Construction of the Continuous Distillation Column

A continuous distillation column was prepared using readily accessible laboratory glassware. This prototype system was assembled from interconnected Hemple distilling columns (24/40 joint), a 250 mL three necked RBF, and a Dean-Stark tube. The column was filled with 0.16″ aluminum packing (Xtractor Depot, ProPack). The joints were sealed with high vacuum grease (Dupont, Molykote). A condenser was placed above the Dean-Stark tube to condense the vapors. The condenser was chilled with process water to 5 °C using a Julabo recirculating chiller. The RBF was heated with a heating mantle to ~  82 °C for binary distillation experiments and continuous distillation of the process stream. Resistive temperature devices (RTD’s) were lined vertically along the outside of the column to monitor temperature. The temperature sensors were encased within mineral wool to maintain temperature across the height of the column.

During continuous distillation, the process stream was introduced to the upper region of the column via a Vapourtec SF-10 peristaltic pump. The pump was calibrated to deliver feed rates ranging from 1–10 mL/min through peristaltic grade tubing. The process stream was pumped to the column at 2–3 mL/min for engineering test runs. The reflux ratio was controlled by a Raspberry Pi controller and a pneumatic air valve which directed aliquots of condensate either back to the column (*i.e.*, reflux, L) or to a waste vessel (*i.e.*, distillate, D). The reflux ratio, R_D_, is defined as the volumetric ratio of the reflux (L) flowrate to distillate (D) flowrate. R_D_ was set by the controller to values ranging from zero to four. To alter R_D_, the pneumatic valve was coded to collect aliquots of sample in timed intervals (T_L_ and T_D_) and directed accordingly. After each time interval, the valve was triggered to direct the next liquid aliquot either to reflux or the distillate. The bottoms product was collected using a peristaltic pump to remove sample from the RBF at an average flowrate of ~ 1.5 mL/min. The flowrate exiting the bottoms vessel was controlled by a feed forward controller to maintain height within the column while preventing the level from dropping or flooding from occurring.

### Process Automation

The primary automation components include (I) temperature monitoring, (II) reflux control, and (III) level control of the distillate and bottom vessels. Automation of the distillation column was achieved by utilizing a Raspberry Pi (5 Series, 8 GB RAM) as a microcontroller for reflux ratio control. Temperature monitoring was enabled by connecting an Arduino-style microcontroller (Elegoo, 2560 MEGA R3) to a series of resistance temperature detectors (RTD’s). Lastly, level control was facilitated by connecting a Raspberry Pi to a four channel Ismatec Reglo ICC peristaltic pump to and analytical balance (A&D, EK6100i). Temperature monitoring was enabled by connecting an Arduino-style microcontroller (Elegoo, Mega R3) to a series of RTD’s while data was collected in and graphed with a Python code. A PC was connected to the Elegoo via a USB hub. Next, four RTD’s (T-Pro, PT100) were connected to a series of breakout boards (Play-with-Fusion, MAX31865 PT100) and were mounted onto a breadboard. The breadboard was connected to the microcontroller using Dupont cables. The wires were positioned along the vertical height of the distillation column, between the insulation and the column.

Reflux control was enabled by utilizing a Raspberry Pi 5 Series and a Python automation code to periodically direct the condensing liquid either back to the column (reflux) or to a waste vessel (distillate mode). Set point control of the Reflux ratio (R_D_ = L/D) was achieved by actuating a valve to direct the flow at set time intervals (T_L_ and T_D_). The Raspberry Pi was connected to a single channel relay switchboard (Songle, SRD-5VDC-SL-C) via breadboard cables (Dupont). The relay was subsequently connected to a pilot valve (SMC Pneumatics, SYJ314-5HZ-M5) which directed on/off airflow to a pneumatic valve (SMC, LVA200-02N-C). The pneumatic valve was located along the base of the Dean-Stark tube and connected to tubing to provide directional control of the condensing liquid. During operation, house air was constantly fed to the pilot valve at 40–50 psi while the pilot valve remained in a closed and gated position. While in this state, the pneumatic valve was left open and liquid flow was directed towards reflux. Upon actuation of the pilot valve by the Raspberry Pi, airflow was delivered to the pneumatic valve to divert the condensate to the distillate vessel through PTFE tubing. The distillate vessel consisted of a 250 mL Erlenmeyer flask.

Level control was enabled via feed forward control of the distillate and bottom’s vessels. The distillate vessel was placed on an analytical balance (A&D, EK-6100i) to record the mass of distillate recovered from the automated reflux system. A four-channel peristaltic pump (Ismatec, Reglo ICC) was used to recover the product stream out from the bottom’s vessel using 3-stop Viton tubing (2.06 mm ID). Replacement solvent was then added to the bottom vessel through the same pump in order to restore the alcohol which was stripped off during distillation. Automation was enabled using a Raspberry Pi 5 which was connected to the pump via a USB Mini-B to USB converter and to the scale using a RS232 to USB converter. A Python code was developed to periodically record the mass of the distillate vessel. The flowrate entering the distillate vessel (D) was then calculated (g/min) and converted to a solvent factor (e.g., S = 0.25 × D for IPA) which was based upon the amine-to-solvent density ratio in order to determine the necessary amount of solvent to be replaced. The overall mass balance equation was given by the following equation:

$${M}_{Bottoms}=\left({M}_{Feed}+{M}_{S}\right)-{M}_{Distillate}$$

### Analytical Characterization

High performance liquid chromatography (HPLC) was utilized by employing an Agilent 1200 with an Eclipse XDB-C18 column (5.0 m; 4.6 µm × 250 mm) and a diode array detector (DAD). The eluting solution was scanned at a wavelength (λ) of 220 nm. Samples were prepared for HPLC analysis by filtering (200 µm) the sample and diluting with methanol in a volumetric flask to a target concentration (1–4 mg/mL). Calibration curves were prepared using commercial standards. Concentration was determined via peak integration and comparison to the calibration curve for accurate chemical assay. The mobile phase gradient consisted of 0.1% phosphoric acid in water and HPLC grade methanol. The HPLC method used a mobile phase of 85% H_3_PO_4_ and 15% MeOH at Q = 1.7 mL/min. The column temperature was set to 30°C.

Gas chromatography flame ionization detection (GC-FID) was performed using an Agilent 7000 GC. The column consisted of a Restek Rtx-Volatile Amine column (L 30 m x ID 320 µm x FT 5 µm). UHP Grade helium was used as the carrier gas at 2.0 mL/min and a 10:1 split ratio. The oven was ramped at 2°C/min from 60–70°C. GC-FID calibration curves were generated using calibration standards consisting of TBA and alcohol mixed in acetonitrile (ACN) with concentrations ranging from 0.5–5.0 mg/mL. The injections were made in triplicate when generating the calibration curves.

## Results

### Process Chemistry – Continuous Albuterol Sulfate Manufacturing

**Fig. 3:**
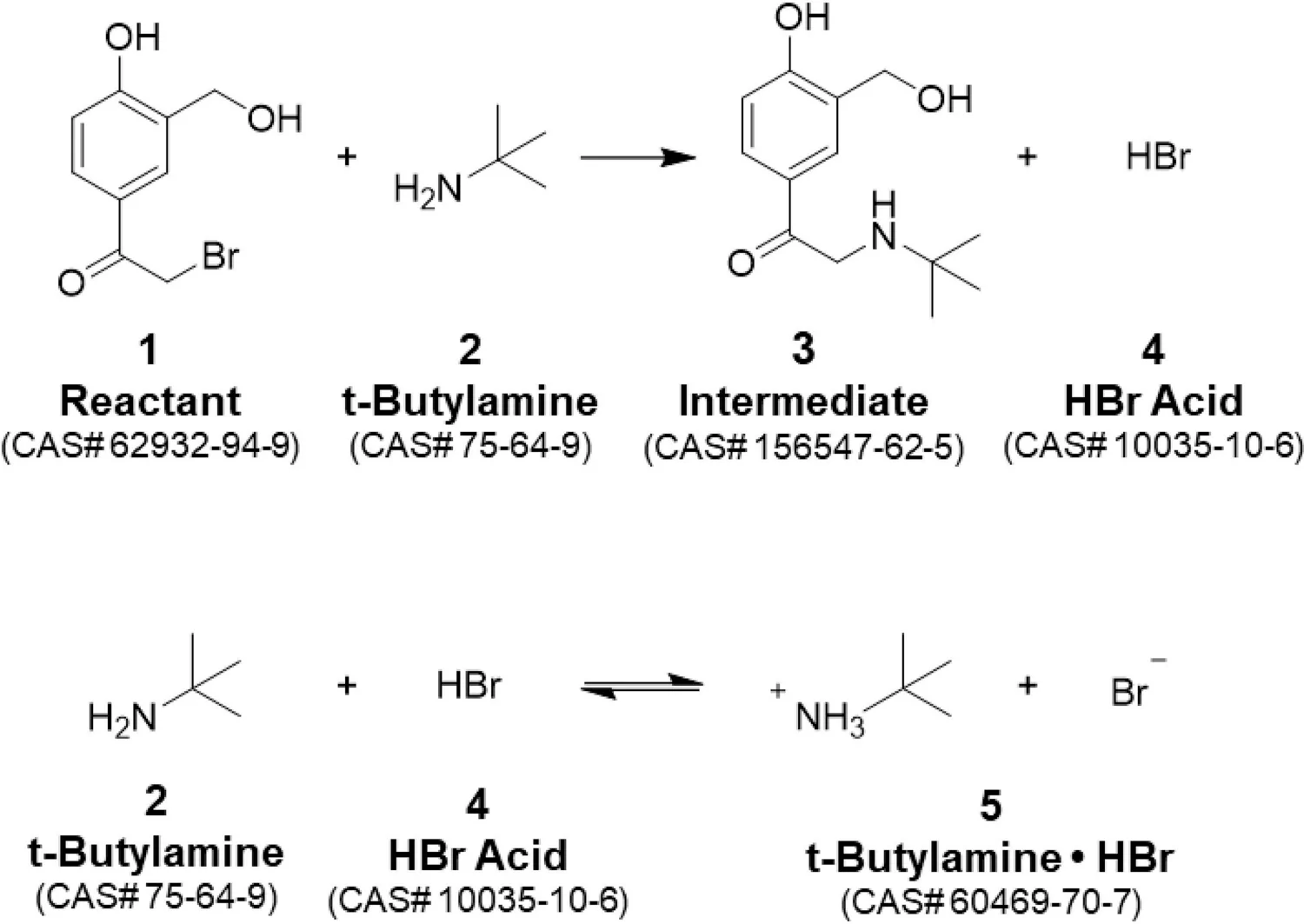
The S_N_2 amination chemistry performed in the LFR unit operation located directly upstream from the distillation column.

The overall process chemistry and process flow diagram (PFD) are provided in the supplementary information section (See SI Figures S1-S2). The first unit operation in the system involves the S_N_2 amination of a bromo-diol starting material with tert-butylamine to form a key aminated intermediate; see Fig. 3 [11]. The process is conducted within a laminar flow reactor (LFR) at a SM concentration of 50 mg/mL. As previously reported, this synthetic process enabled 100% conversion and > 90% yield when performed in common alcohol solvents (*i.e.*, MeOH, EtOH, and IPA) at 60°C. This reaction occurs immediately upstream from the distillation column, and the reaction contents are then pumped into the distillation unit for separation.

In order to drive the upstream reaction to completion, four equivalents of TBA (**2**) are utilized for each equivalent of the bromo-diol starting material (*i.e.*, SM, molecule **1**). Upon amination of the SM to form the key intermediate (*i.e.*, molecule **3**), an acidic HBr byproduct (4) is released which gradually lowers the pH and would eventually quench the reaction without the incorporation of additional base. Thus, the upstream S_N_2 amination reaction is run in four molar equivalents of TBA per SM. The excess TBA functions as a weak Lewis base to scavenge the excess protons and form a TBA·HBr salt (Molecule 5). The overall process chemistry is specified in Eqs. 1 and 2.

1 $$SM+4TBA\to API\; Intermediate +3TBA+HBr$$

2 $$TBA+HBr\rightleftharpoons TBA\bullet {H}^{+}+{Br}^{-}$$

The main amination byproduct consisted of small amounts of dimerized intermediate (See SI Fig. S4, < 5% dimer) while the major impurity on a concentration basis consisted of the unreacted amine [11]. The dimer impurity is structurally similar to the desired intermediate and is therefore not expected to be selectively removed by distillation due to its high boiling point and decomposition temperature. Rather, the purpose of the distillation operation is the removal of volatile TBA while retaining the intermediate within the liquid phase. While thermal degradation of the aminated intermediate could theoretically increase the relative concentration of impurities, the operating temperatures employed by the distillation column were well below the decomposition temperature of key analytes. HPLC analysis did not demonstrate the increase of impurities due to thermal decomposition. Furthermore, the downstream hydrogenated intermediate possesses greater thermal stability than the aminated intermediate, reducing concerns regarding impurity enrichment during subsequent processing.

Here we demonstrate the removal of TBA via in-line distillation at a mid-point within the manufacturing process, while final purification is achieved by using the Nutsche filter dryer to remove the dimer and any remaining trace impurities (See Fig. 1). The distillation column was positioned immediately downstream from the amination reactor to remove TBA prior to catalytic hydrogenation and API crystallization as this location helps prevent downstream catalyst deactivation and streamlines the final crystallization and filtration steps. For effective removal of TBA by distillation, the selected process solvent should have a boiling point that differs substantially from that (*i.e.*, ΔT_Bp_). Additionally, the mixture should possess a large relative volatility (αᵢⱼ) between component *i* (i.e., the light-boiling component) and component *j* (i.e., the heavy-boiling component) to aid separation. Here, *y* is the composition of the vapor phase and *x* is the composition of the liquid phase [27]. Lastly, a desirable process solvent consists of one which will not form an azeotrope with the amine (*α* ≠ 1.0). Azeotropes are difficult to separate as the composition is the same in both the liquid and vapor phase (*i.e.*, y_i_ = x_i_ and y_j_ = x_j_).

3 $${\alpha}_{ij}=\frac{\left(\frac{{y}_{i}}{{x}_{i}}\right)}{\left(\frac{{y}_{j}}{{x}_{j}}\right)}=\frac{{K}_{i}}{{K}_{y}}$$

Key distillation parameters for TBA, the intermediate (**3**), and select process solvents were obtained from NIST and ASPEN + (See Table 1) [31]. The temperature difference between the boiling points of the solvent and amine were calculated for comparison (*i.e.*, ΔT_Bp_ = B_P_^ROH^ – B_P_^TBA^). The largest temperature difference is observed between TBA and isopropanol (ΔT_Bp_^IPA^ = 37.4°C), suggesting that IPA would be the best process solvent for the distillation unit operation. Conversely, methanol provided the smallest difference between boiling points (ΔT_Bp_^MeOH^ = 19.7°C), while the ΔT_Bp_ between the TBA and the intermediate (*i.e.*, ΔT_Bp_^Int.^ = 331.8°C) is sufficiently large to prevent volatilization of the intermediate at process conditions (*i.e.*, T_Process_ <  100 °C). Additionally, the relative volatility between IPA and TBA was highest in comparison to the other solvents (*α*_*TBA,IPA*_ = 2.76). This is a key aspect that lends to the effectiveness of distillation for impurity removal.

**Table 1:** Comparison of key parameters for process chemicals including molecular weight (Mw), boiling point (B_P_), boiling point difference (ΔT_Bp_), and relative volatility (α_ij_ = K_i_/K_j_)

Chemical	Mw [g/mol]	Bp [°C]	ΔT_Bp_ (B_p_^j^ –B_p_^TBA^)	Relative Volatility (α_ij_) (K_i_ = TBA, K_j_ = ROH)
Tert-butylamine	73.14	45.0	-	-
Methanol	32.04	64.7	19.7°C	1.70
Ethanol	46.07	78.4	33.4°C	2.49
Isopropanol	60.09	82.4	37.4°C	2.76
Albuterol Intermediate	237.29	376.8	331.8°C	-

*Parameters are based upon ASPEN + simulations at process conditions as calculated via the NRTL model. Additional detail is provided in the methods section

While vapor–liquid equilibrium behavior and relative volatility are important considerations for distillation design, solvent selection in pharmaceutical manufacturing must also consider residual solvent limits, safety, environmental impact, solvent recovery, and downstream processing requirements. Both ethanol and isopropanol are classified as ICH Class 3 solvents and are generally regarded as having lower toxicological concern than methanol, which is classified as an ICH Class 2 solvent with more stringent residual-solvent limits. Additionally, recovery and recycling of the process solvent may improve process economics and reduce solvent consumption during continuous operation. Therefore, solvent selection for the distillation operation was based not only on separation performance, but also on broader process development considerations relevant to pharmaceutical manufacturing. Residual process solvents are subsequently reduced during downstream isolation and drying operations, including the automated Nutsche filter-dryer shown in Fig. 1.

### Binary Distillation and Process Simulation in ASPEN + 

**Fig. 4:**
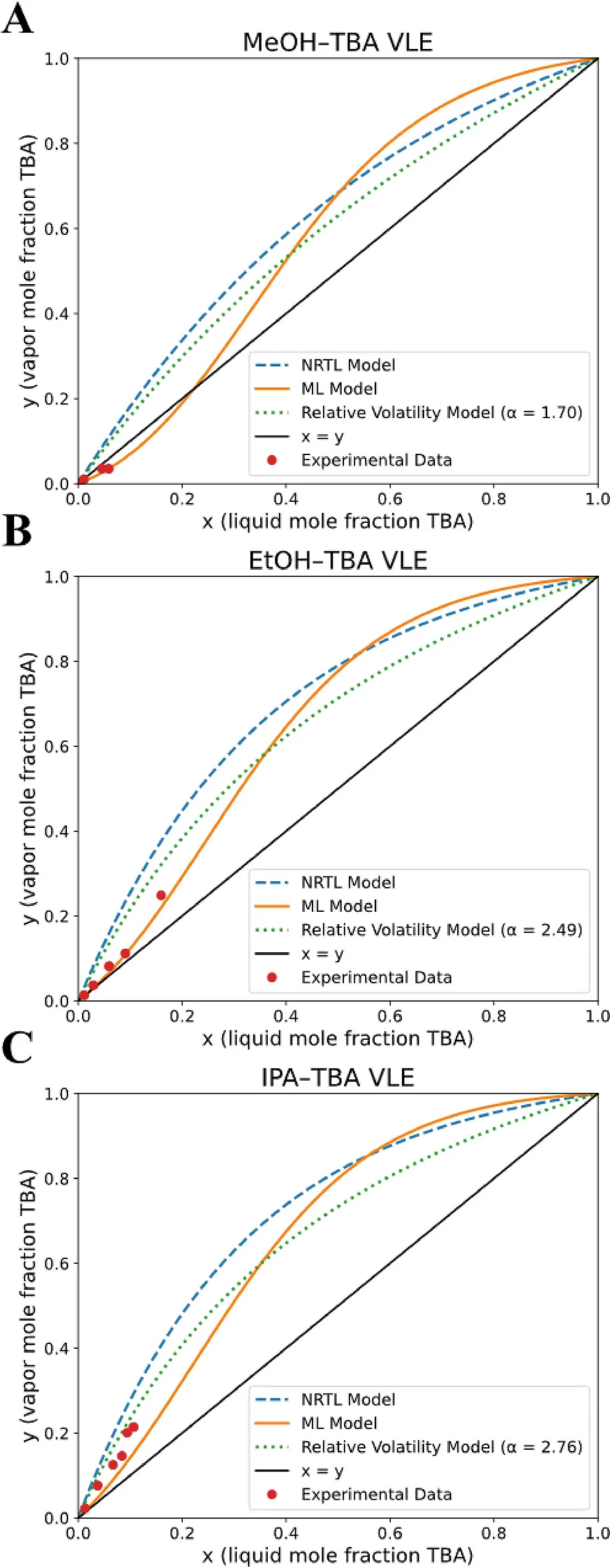
VLE curves for binary process mixtures *versus* experimental data. Mixtures include (**A**) TBA-MeOH, (**B**) TBA-EtOH, and (**C**) TBA-IPA. Data was simulated in ASPEN + using the NRTL and Relative Volatility model. The EquiNet model was simulated in Python.

Binary vapor liquid equilibrium curves were simulated in ASPEN + for each TBA-Alcohol mixture (e.g., TBA-MeOH, TBA-EtOH, and TBA-IPA) to assess the phase envelope, potential for azeotrope formation, and prospects for separation. This process was first performed assuming each solution acts as an ideal mixture according to Raoult’s Law (See Eq. 4, and SI Fig. S5). Next, the mixtures were simulated to account for non-idealities using the relative volatility model, the NRTL model, and the neural network-based EquiNet model (See Fig. 4). Experimental data were collected for binary mixtures of each process solvent to determine which model best fit the experimental data. Finally, an optimal process solvent was selected for continuous distillation.

4 $${p}_{i}={x}_{i}{p}_{i}^{0}\left(T\right)$$

Raoult’s Law enables the calculation of equilibrium phase behavior of mixtures based on their pure component vapor pressures: P_i_^0^. Here the vapor pressure is given by the liquid mole fraction (*x*_i_) multiplied by its pure component vapor pressure. This model assumes the mixture acts as an ideal mixture and is given by the Antoine equation (See Eq. 5), where a, b, and c consist of empirically obtained constants unique to each molecule. VLE curves as calculated via Raoult’s law predict idealized mixture parameters and present characteristic bow-shaped VLE envelopes as shown in SI Fig. S5. However, mixtures do not always exhibit ideal behaviors; thus, more advanced models and experimental methods are required.

5 $$log\left({P}^{sat}\right)=a-\frac{b}{T+c}$$

Non-ideal mixing phenomena, such as azeotropes, can form depending on the unique functional groups present on each molecule and their tendency to form molecule-to-molecule bonding via hydrogen and Van der Waals forces. To correct for mixture non-idealities, an activity coefficient (γ_i_) was added and the VLE equation to correct for non-ideal behavior (See Eq. 6). The non-random two-liquid model, *i.e.*, the NRTL model, was utilized to simulate the mixtures as presented in Fig. 4. Finally, the neural network EquiNet model was utilized for comparison to binary VLE data.

6 $$P{y}_{i}={P}_{i}^{sat}{\gamma}_{i}{x}_{i}$$

The simulated VLE curves for each binary mixture are provided in Fig. 4A-C. Both the NRTL and relative volatility model generally predicted ideal mixture behavior as noted by the characteristic bow-shaped curvature protruding outward along the VLE curve *versus* the x = y reference line. Conversely, the EquiNet model predicted slight azeotropic behavior for all binary component mixtures as noted by the vicinity of the EquiNet’s VLE curve to the x = y reference line and its “s-shaped” curvature which is characteristic of binary azeotropes.

As predicted based upon the data provided by Table 1, the VLE curves for the TBA-IPA mixture indicated the largest potential for separation as noted by the significant bowed curvature and wide envelope formation (See Fig. 4C). Conversely, the TBA-MeOH mixture demonstrated poor separation potential as noted by the relative proximity of the TBA-MeOH VLE curve to the reference line. Finally, the TBA-EtOH VLE curve provided only moderate separation potential. Next, Txy-diagrams were simulated in ASPEN + for each mixture to assess the phase envelope of TBA in each solvent (See SI Fig. S5). Again, the TBA-IPA mixture presented the largest phase envelope, suggesting a greater propensity for separating TBA.

The experimental VLE data is plotted in Fig. 4 (red data points) for comparison of experimental data *versus* each VLE model. In the case of the TBA-MeOH mixture the experimental data appeared to track the EquiNet model the best, indicating the presence of an azeotrope at low mole fractions of TBA. However, the TBA-EtOH and TBA-IPA mixtures both showed more idealized behavior as each mixture nicely tracked the relative volatility model. Thus, the TBA-IPA solution was selected for further continuous distillation studies due to the large ΔT_Bp_, its large relative volatility, and its resistance to azeotrope formation.

### Continuous Distillation Process Overview

After identifying an optimal process solvent (*i.e.*, IPA), a series of prototype distillation columns were constructed to test the effectiveness of continuous in-line distillation for the removal of TBA from the process stream. Initial distillation columns consisted of benchtop configurations which provided moderate potential for TBA removal (See SI Fig. S6) while larger prototypes were developed for setpoint optimization. Process material was produced upstream using a Vapourtec flow reactor to synthesize the key albuterol intermediate (3) in IPA (See Fig. 3). The process stream was subsequently pumped into the top of the distillation column at 2–3 mL/min (*i.e.*, a stripping column configuration) and the concentration of TBA was quantified in each stream via off-line GC-FID (*i.e.*, the feed stream (F), distillate stream (D), and bottoms stream, (B)). The intent of the stripping configuration was to remove TBA from the bottoms stream while collecting it in a waste distillate stream. The bottoms stream could then be delivered to a downstream catalytic reactor for production of the final API.

In order to improve TBA separation performance, the height of the distillation column was extended, and additional packing material was added to create a prototype + 3-foot pilot-scale distillation column. This pilot-scale system was equipped with a Dean-Stark tube and automated with a reflux control system using in-house Raspberry Pi microcontrollers. The column was simulated in ASPEN + to aid in column engineering and process optimization to achieve improved TBA removal (See Methods). ASPEN + was used as a process-development and screening tool to evaluate operating trends, parameter sensitivity, and identify conditions for experimental investigation. Representative input and output parameters are presented in Table 2 for the experimental column *versus* ASPEN + simulation results using the NRTL model. The output parameters generally track those of the experimental results for a 2–3 mL/min feed basis. However, subsequent experimental studies identified operating behavior, particularly with respect to reflux ratio, that was not fully captured by the simulation. Additional simulations are presented in the SI (Table S2).

**Table 2:**
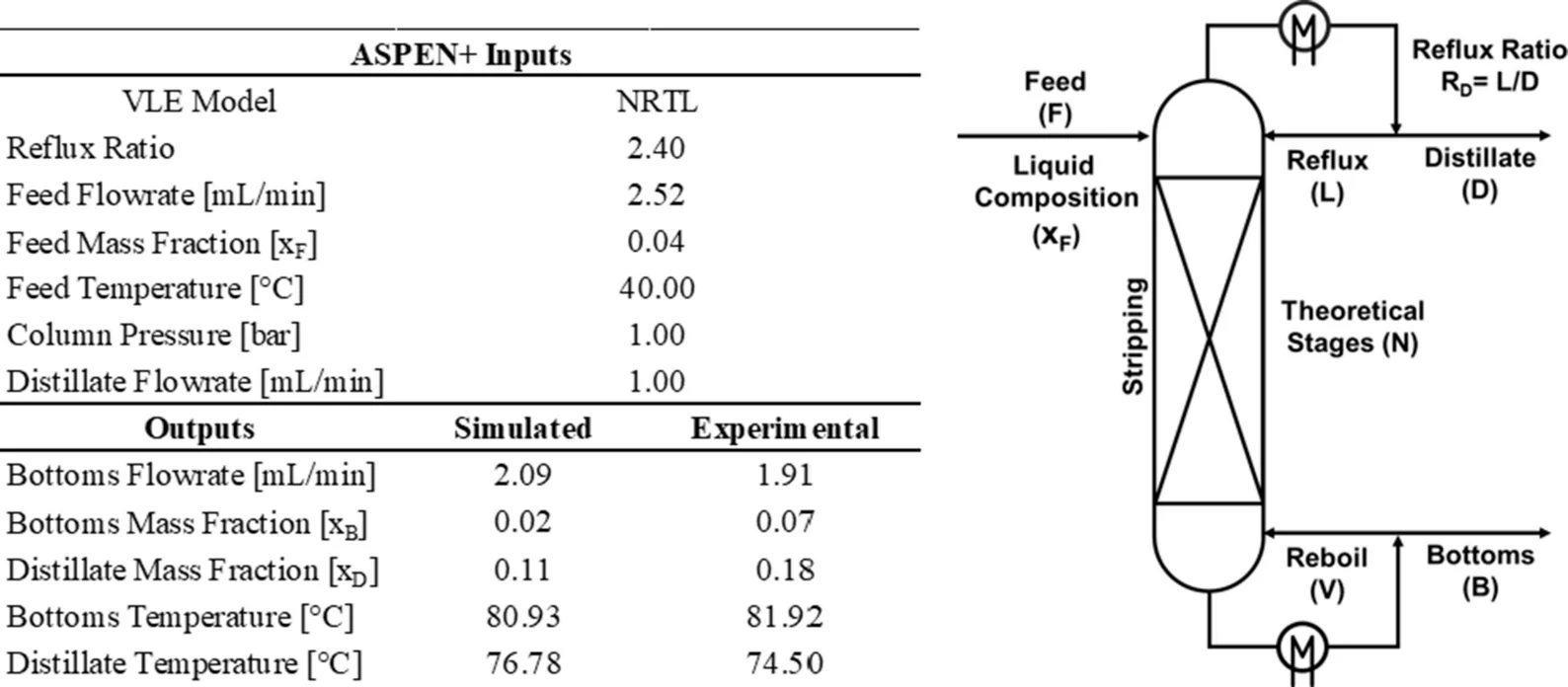
Parameters for simulation of a continuous distillation column in ASPEN +

**Fig. 5:**
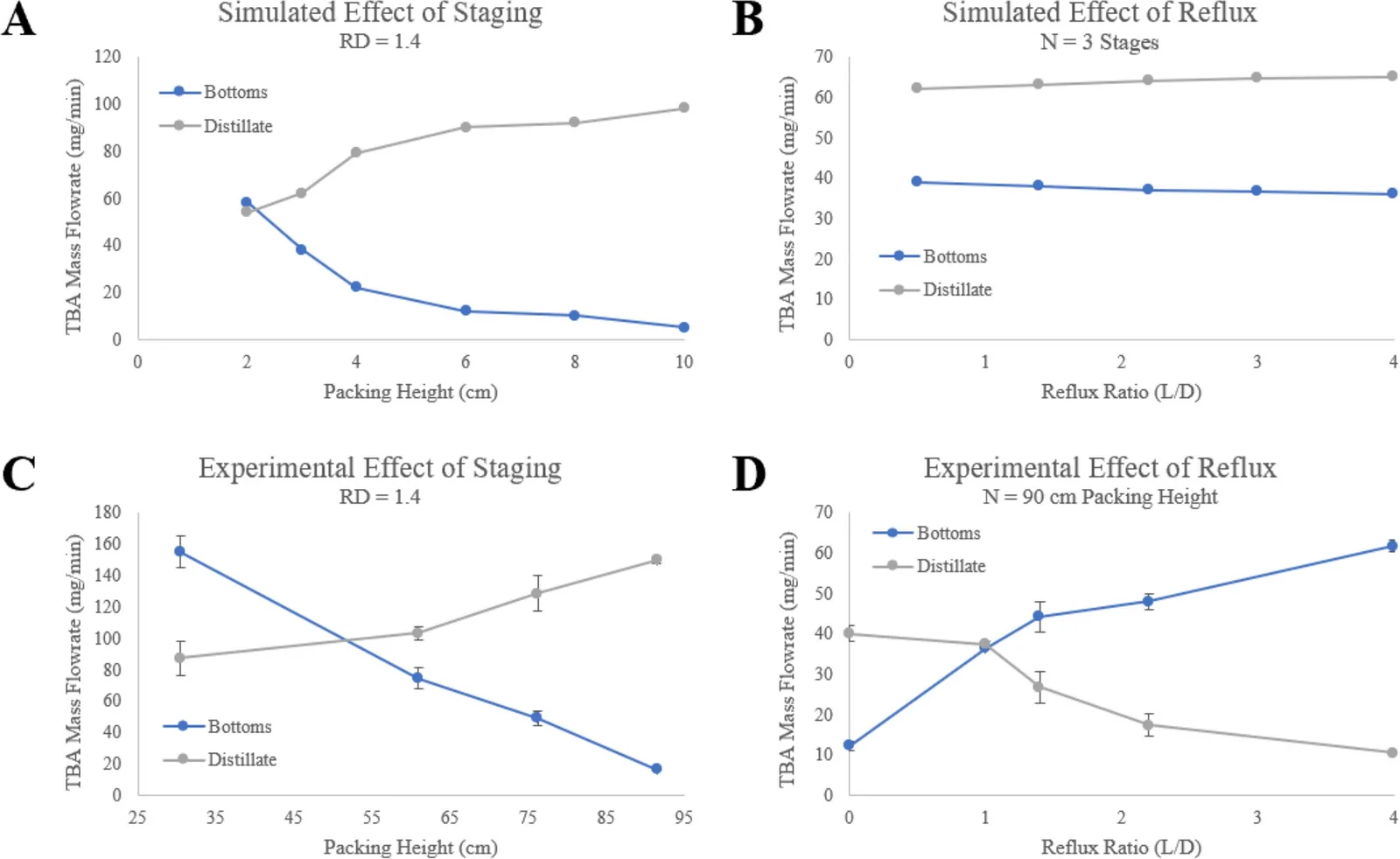
Comparison of simulated and experimental TBA separation performance as a function of packing height and reflux ratio. (**A**) ASPEN + simulation of the effect of packing height on TBA mass flowrate at a constant reflux ratio (L/D) of 1.4. (**B**) ASPEN + simulation of the effect of reflux ratio on TBA mass flowrate at a constant 3 theoretical stages. (**C**) Experimental effect of packing height on TBA mass flowrate at a constant reflux ratio (L/D) of 1.4. (**D**) Experimental effect of reflux ratio on TBA mass flowrate at a constant packing height of 90 cm.

The effect of reflux ratio (RD = L/D) was simulated to evaluate the influence of reflux ratio on the pilot-scale distillation column (See Fig. 5). The results show the TBA mass flowrate in the distillate and bottoms streams as calculated from simulated outlet flowrates and corresponding TBA mass fractions under experimental feed conditions. Increasing the reflux ratio was observed to produce only a modest shift in TBA composition in the distillate (See Fig. 5A). However, the ASPEN + simulations demonstrated a large propensity for TBA separation when increasing the stage number (See Fig. 5B). Thus, the simulation suggested that distillation performance can be enhanced by adding height to the column and providing additional packing material (or stages) to provide ample contact points for separation. The limited benefit of reflux predicted by the ASPEN + simulations is attributed to the relatively large volatility difference between TBA and IPA under the operating conditions investigated. Consequently, the simulations suggested that additional equilibrium stages would provide a greater improvement in separation performance than increasing reflux ratio alone.

Continuous distillation was then demonstrated in the pilot-scale system for comparison with the ASPEN + simulation. The reflux ratio was systematically varied from zero to four by actuating a pneumatic valve to recycle the stream (*i.e.*, reflux) or divert the stream to waste (*i.e.*, distillate collection). The TBA composition was monitored with off-line GC-FID to quantify the TBA mass flowrate in the distillate and bottoms stream (See Fig. 5C). Contrary to the ASPEN + predictions, increasing reflux resulted in an undesirable increase in TBA concentration within the bottoms stream and reduced overall TBA removal efficiency (See Fig. 5C). The best experimental performance was observed in the absence of reflux (R_D_ = 0), indicating a significant discrepancy between the simulated and experimental behavior of the system. The effect of packing height, however, matched that predicted by the ASPEN + simulation and enabled the removal of TBA when the height of the packing was increased (See Fig. 5D).

Although the measured temperature difference between the top and bottom of the column was relatively small (~  3 °C), both the ASPEN + simulations and experimental packing-height studies demonstrated that separation performance remained highly dependent on the availability of vapor–liquid contact stages. Thus, separation within the column was driven primarily by repeated vapor–liquid equilibrium interactions between TBA and IPA rather than by a large axial temperature gradient.

**Fig. 6:**
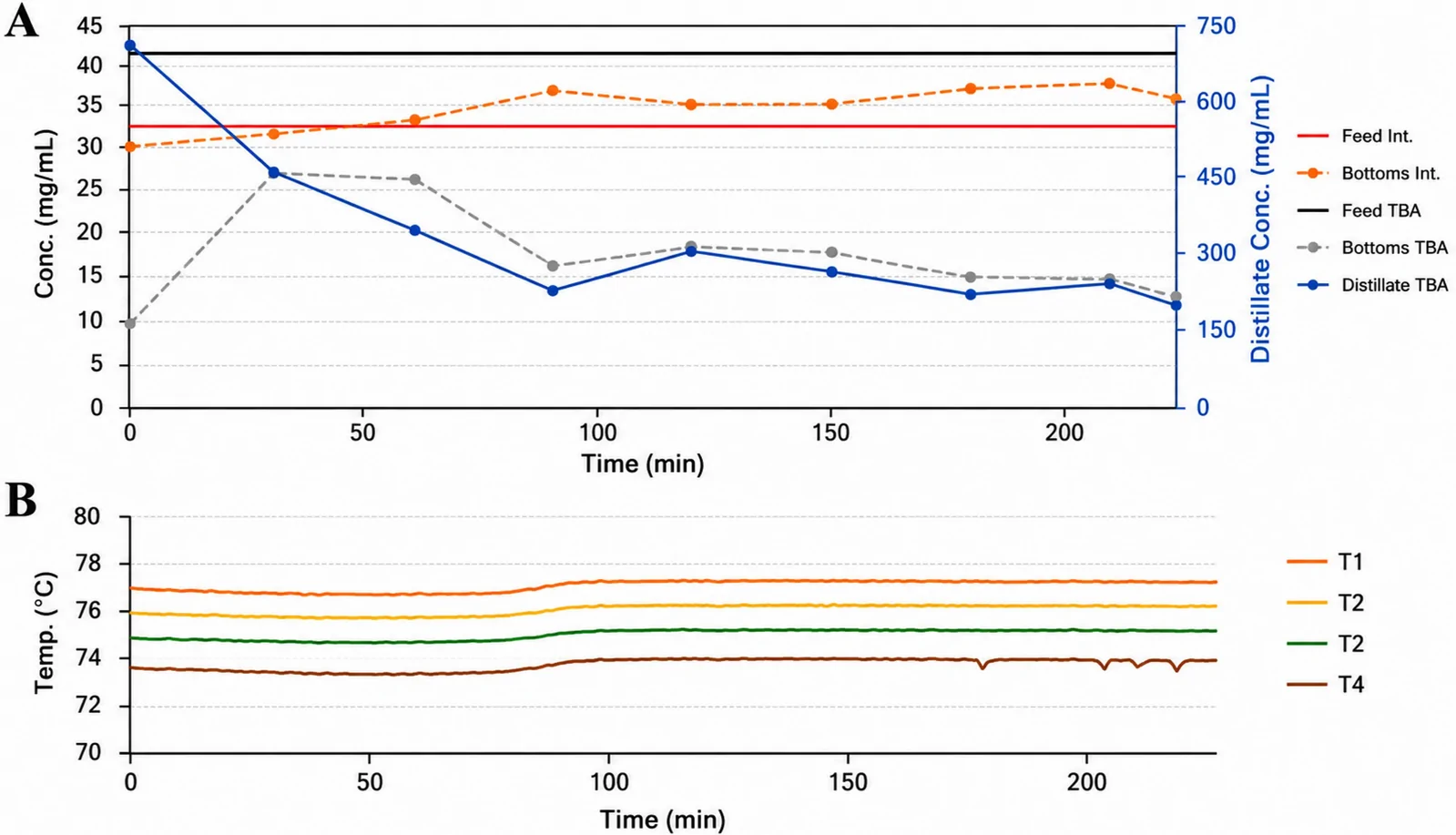
Time-on-stream data obtained during continuous distillation of an albuterol intermediate process stream. The stream contains as-synthesized albuterol intermediate (**3**) and excess TBA in IPA. Data includes (**A**) concentration of albuterol intermediate and TBA in the feed, distillate, and bottoms stream as measured with HPLC and GC-FID; (**B**) on-line temperature measurement during distillation as recorded by the temperature sensors located at the bottom, mid-bottom, mid-top, and top of the column.

Extended continuous distillation experiments were performed to assess time-on-stream performance for TBA removal from authentic albuterol intermediate process streams (See Fig. 6). Temperature sensors were placed along the column to monitor temperature, while the entire system was thermally insulated to maintain constant VLE (See SI Fig. S6). The thermally insulated system was able to maintain a constant temperature profile while simultaneously reducing the TBA concentration within the process stream. These conditions were achieved over the course of + 3 h’ time-on-stream. The concentration profiles observed during continuous operation are shown in Fig. 6A. Throughout the time-on-stream study, the concentration of the albuterol intermediate remained relatively constant in both the feed and bottoms streams, indicating minimal loss of intermediate during distillation. Simultaneously, TBA concentrations in the bottoms stream remained substantially below the feed concentration, demonstrating sustained removal of the volatile amine impurity during continuous operation. Robust temperature control was maintained across the height of the column as demonstrated by Fig. 6B where the bottoms temperature was held constant at 78.5 ± 0.5°C while the top of the column was maintained at 75.5 ± 1.0°C. These results suggest that the distillation column maintained stable separation performance over the duration of the experiment.

The continuous distillation system was run in the absence of reflux and enabled removal of approximately 75% of the GC-detectable TBA present in the process stream as assessed via GC-FID. The upstream amination reaction was charged with 60 mg/mL TBA (*i.e.*, 4 molar equivalents relative to the starting material). Following reaction, the stream entering the distillation column contained approximately 45 mg/mL TBA, corresponding to approximately 3 molar equivalents relative to the intermediate. Distillation reduced the TBA concentration to approximately 10 mg/mL in the bottoms stream. This corresponds to an inlet TBA feed concentration of 8.0 mol% in IPA (*i.e.*, 45 mg/mL) which was reduced to 0.5 mol% (*i.e.*, 10 mg/mL).

The residual TBA concentration observed after distillation likely reflects pH-dependent speciation between volatile free-base TBA and protonated TBA present as the corresponding TBA·HBr salt. As free TBA is removed during distillation, the liquid phase becomes less basic, shifting additional amine toward the less volatile protonated form and limiting complete TBA removal. The resulting bottoms stream product was subsequently hydrogenated over a catalyst to form albuterol freebase in accordance with the end-to-end process flow diagram shown in Fig. 1. Any remaining impurities are removed via a final automated Nutsche filter dryer to reach FDA impurity guidelines [13]. Collectively, the successful removal of TBA during continuous operation, stable temperature profiles throughout the column, and retention of the desired intermediate within the bottoms stream demonstrate the feasibility of integrating this distillation operation within a larger advanced manufacturing technology platform. Thus, this continuous distillation prototype successfully demonstrated the proof-of-concept data necessary for translation to a candidate advanced manufacturing technology.

## Discussion

In this work, we demonstrated the application of continuous distillation for removal of excess tert-butylamine during the end-to-end continuous synthesis of albuterol sulfate. The current system is under construction within an advanced manufacturing pilot plant for cGMP manufacturing at a contract manufacturing organization (CMO). The following discussion highlights the engineering design considerations and integration principles identified during development of this in-line continuous distillation system.

The functionalization of drug intermediates with nitrogen-based pharmacophores is a key synthetic step during the synthesis of drug molecules and is one of the most common transformations in the pharmaceutical industry [32]. Nitrogen-based functional groups retain a lone pair of electrons which can selectively bind to receptors within the human body to provide therapeutic effects [33]. This lone pair of electrons can selectively react with leaving groups (e.g., Br^−^ and Cl^−^) through S_N_2 amination reactions to functionalize the API’s carbon backbone. This process typically releases a proton from the amine and a negatively charged halide ion, a result that effectively increases the acidity of the reaction solution [34]. Next, nearby unreacted amines can act as a weak Lewis base to scavenge the newly released protons to form amine salts (e.g., TBA·HBr).

S_N_2 amination reactions are often run in excess molar equivalents to help drive the reaction to completion. However, the excess reactant must be removed from the product stream to ensure drug quality. Small molecule amines often lack chromophores, making them difficult to detect using UV diode array detectors [11]. Additionally, once protonated to their corresponding ammonium salts, they lose the volatility required for removal by distillation, necessitating alternative downstream purification strategies. Fortunately, however, many small molecule amines are lightweight and volatile, a property which enables their removal via distillation. Conversely, APIs and their corresponding intermediates are often much larger in mass and possess high boiling points. Thus, the incorporation of in-line distillation columns for drug substance purification has the potential to become a key emerging technology in the coming years. Continuous in-line distillation is advantageous if the following criteria are met.

Criterial for Continuous In-Line Distillation:

I. A large ΔT_Bp_ exists between the excess reagent (e.g., amine) and process solvent.
II. A large relative volatility exists between the reagent and solvent (*α*_*ij*_ >  > 1.0).
III. The process solution does not form an azeotrope (*i.e.*, *α*_*ij*_ ≠ 1.0, y_i_ ≠ y_j_, x_i_ ≠ x_j_).
IV. The API or intermediate has a high boiling point T_Bp_^API^ >  > T_Bp_^i^.
V. The API or intermediate is not susceptible to thermal degradation.

A potential use case was identified within this text for continuous in-line distillation based upon the use of a molar excess of a key reagent (*i.e.*, tert-butylamine, TBA) to synthesize albuterol.. This article uniquely demonstrates how one can remove large quantities of a volatile impurity (*i.e.*, TBA) which is in significant excess of a SM via distillation (*i.e.*, TBA is introduced in 300% excess of the SM). A crystallization tank is used downstream to form the final API salt (*i.e.*, the drug substance, albuterol sulfate), while an automated Nutsche filter dryer is included at the end of the process to rinse the API filter cake and remove any trace impurities (*i.e.*, larger oligomers, < 5%, and trace salts). A primary reason for the location of the distillation column is to remove the excess TBA equivalents such that they do not form TBA salts upon addition of H_2_SO_4_ during API formation. This streamlines the purification process within the Nutsche filter dryer to meet FDA specifications.

Key process parameters were obtained from ASPEN + (e.g., Bp, relative volatility etc.) and binary vapor liquid equilibrium curves were simulated for each binary process mixture. TBA possesses a boiling point of ~  45 °C whereas short chain alcohols have comparatively higher boiling points (e.g., Bp^MeOH^ ~ 64.7°C). The large temperature difference between TBA and alcohols indicated that in-line distillation offered a continuous method of removing the excess free TBA from the process stream without suffering API losses traditionally associated with filtration and recrystallization. In this case, the intermediate is a solid when in salt form and possesses a high predicted boiling point Bp^Int^ > 300°C. Thus, the intermediate will not be removed via distillation in this process.

A series of binary flash distillation experiments were conducted to determine the optimal process solvent for distillation. IPA was found to be most suitable as it possesses a high relative volatility in comparison to TBA. As demonstrated via ASPEN + simulations and experimental binary distillation studies, the TBA-MEOH system was susceptible to the formation of an azeotrope. Azeotropes occur when intermolecular functional groups interact to generate non-ideal phase behavior. This often causes the solvents to have similar boiling points and results in uniform composition in both the vapor and liquid phase. Azeotropes are undesirable as they limit the ability to distill a component from the mixture. Next, a custom distillation system was developed for removal of the excess TBA freebase from IPA process streams via continuous in-line distillation. A series of prototype distillation columns were assembled and successful separation of TBA was demonstrated at a process basis of 3.0 mL/min (See SI Fig. S6).

During continuous distillation, key engineering decisions must be considered including the location of the feed stream, the height of the column, the internal configuration (*i.e.*, packing or plates), and the reflux ratio. Fractional distillation columns often include a central feed port located roughly halfway up the column along with rectifying and stripping sections located above and below the feed inlet (See SI Fig. S3). Yet, for select applications it becomes optimal to place the feed port either at the base or top of the column to enable complete rectification or stripping. In the application at hand, the primary goal is to remove the volatile and lightweight TBA impurity from the process stream while the loss of process solvent (e.g., IPA) is acceptable as it can be replaced in the downstream process. Thus, the optimal column configuration for this process consists of a feed port located at the top of the column, near the condenser, and thus establishing a long stripping portion of the column (See SI Fig. S3). In this scenario, the heavy API intermediate possesses low volatility and will easily travel to the base of the column while the lighter weight TBA and alcohol molecules can be readily stripped from the process stream.

In addition to vapor–liquid equilibrium behavior, solubility and fouling risks are important considerations during continuous distillation. As volatile components are removed, the concentration of nonvolatile components in the liquid phase can increase, potentially leading to precipitation, crystallization, or solid accumulation within the column. During IPA-based distillation experiments, an unidentified white crystalline solid was observed primarily within the condenser and overhead regions. Because this solid appeared in the distillate-side portion of the system rather than in the bottoms stream, it was not attributed to precipitation of the nonvolatile albuterol intermediate. No solid accumulation was observed within the reboiler, column packing, or bottoms stream during operation. However, this observation highlights the importance of evaluating solids formation during continuous operation, particularly for overhead regions where volatile amines may concentrate and potentially interact with atmospheric components or trace impurities. Future development should include identification of any accumulated solids, assessment of solvent- and atmosphere-dependent fouling mechanisms, and definition of operating limits that maintain both the intermediate and overhead components in solution.

**Fig. 7:**
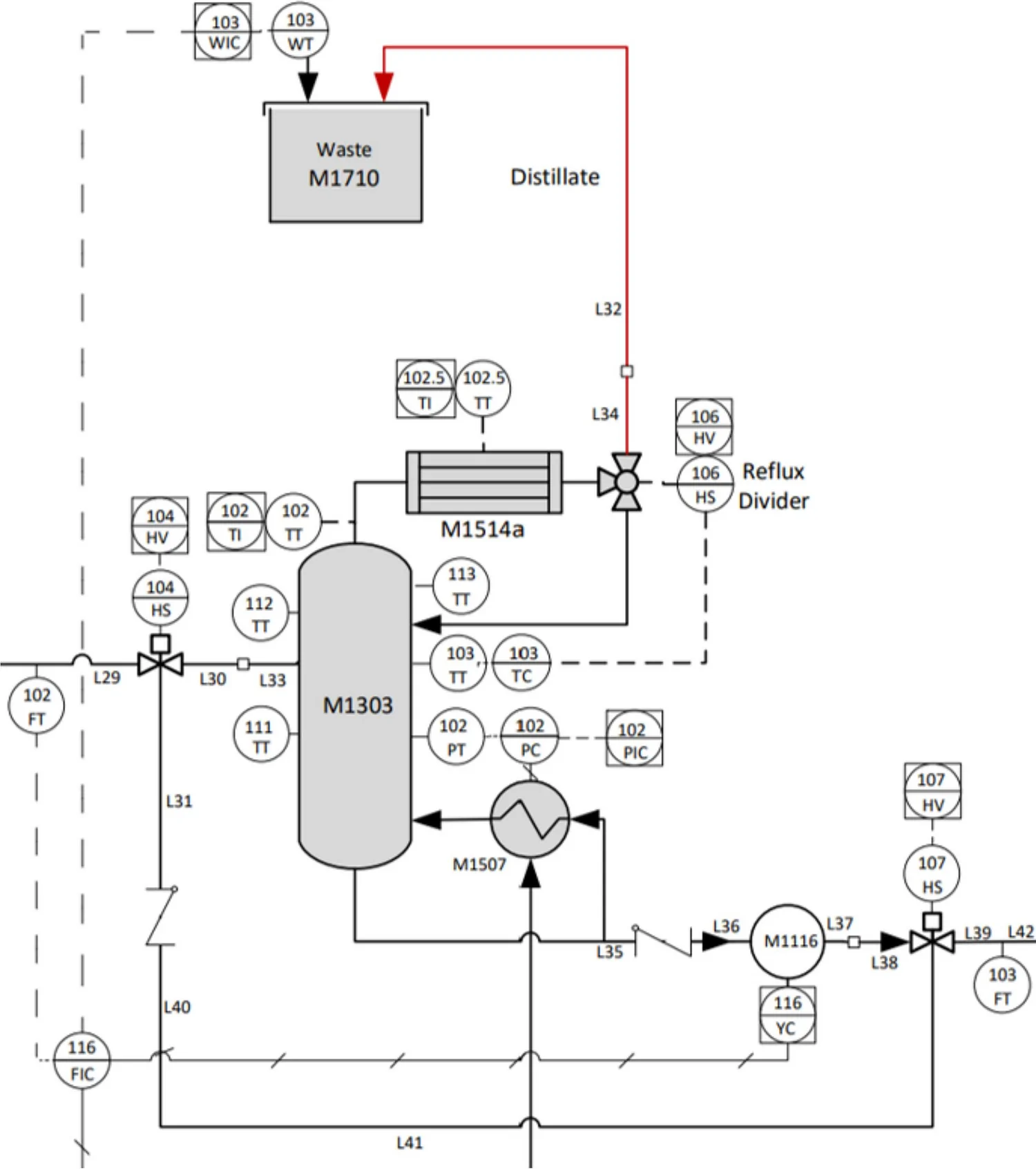
Process and instrumentation diagram (P&ID) of the custom AGI distillation system adapted for continuous albuterol manufacturing. Major equipment, associated functions, and critical process parameters (CPPs) are provided in Table S2 of the Supporting Information.

The final column design is detailed in Fig. 7 for integration within the pilot plant for continuous albuterol sulfate manufacturing. The system consists of a custom blown glass column (AGI) with recessed temperature ports and exterior silvering to monitor and maintain temperature across the height of the column (See Fig. S6C). The column features an Allihn condenser and an optional swinging-bucket reflux divider. The automated reflux control system is actuated via an electromagnet, and the reflux timer is controlled by a distributed control system. The complete piping and instrumentation diagram (P&ID) incorporates a 250 mL reboiler, temperature sensors, and a pressure sensor (Fig. 7), while the major equipment, associated functions, and critical process parameters (CPPs) are summarized in Table S2. The column is packed with Raschig rings to provide ample surface area to enable TBA separation. Critical process parameters (CPPs) are identified throughout the system to ensure adequate TBA removal (Fig. 7; Table S2).

As we have demonstrated in this work, continuous distillation offers a potential avenue towards drug substance purification within advanced manufacturing systems as it enables the continuous on-line removal of volatile organic compounds (e.g., amines). While amines are often key reactant starting materials, they ultimately must be removed to form the final drug substance to meet the FDA’s guidelines for impurity reporting, identification, and qualification [35]. Continuous in-line distillation offers a continuous means of removing impurities at concentrations which compliment traditional separation techniques such as extraction and recrystallization. Ultimately, multiple separation strategies may be necessary to meet FDA impurity thresholds. However, this work provides proof of concept to enable impurity separation via continuous in-line distillation within advanced pharmaceutical manufacturing systems.

## Conclusions

In efforts to mirror the successes achieved in the petrochemical industry for the purification of commodity chemicals, we have developed a continuous distillation system for removal of an impurity from an API process stream. Analogous mini-pharmaceutical refineries can be designed for pilot-scale integration using the strategies identified herein; this strategy includes the simulation of vapor liquid equilibria, laboratory-based parameter screening, distillation prototype development, and pilot-scale integration. Here, chemical property parameters were first simulated in ASPEN + to identify potential process solvents. Multiple equations of state models were employed during the simulation process to assess phase envelopes and to select solvents which avoid azeotrope formation. The EquiNet model provided the best comparison with laboratory data. This simulation suggested that isopropyl alcohol would readily accommodate TBA removal via an in-line distillation column. Next, a prototype continuous distillation column was built with in-house automation solutions to demonstrate proof-of-concept operation during a time on-stream study. Successful separation of the amine was demonstrated when running in continuous mode at a flowrate of 3.0 mL/min. The continuous distillation strategy detailed herein can remove excess TBA during the production of albuterol within an advanced pharmaceutical manufacturing technology. Similar process integration strategies can be replicated using the following general distillation criteria: (I) a large boiling point difference exists between the solvent and impurity, (II) a large relative volatility difference exists between these components, (III) the system does not form an azeotrope, (IV) the chemical products possess high boiling points, and (V) the products have sufficient resistance to degradation at the distillation process temperature. Ultimately, continuous in-line distillation offers a unique separation strategy for the removal of impurities within advanced pharmaceutical manufacturing systems.

## Supplementary Information

Below is the link to the electronic supplementary material.

Supplementary file1 (DOCX 2718 KB)

## Data Availability

Data available upon request.

## Declarations

### Patents

Patents are pending on the technology specified herein.

## The following abbreviations are used in this manuscript:

*AMT*: Advanced Manufacturing Technology
*API*: Active Pharmaceutical Ingredient
*Bp*: Boiling Point
*CAS#*: Chemical Abstract Services Number
*cGMP*: Current Good Manufacturing Practice
*CMO*: Contract Manufacturing Organization
*CSTR*: Continuously Stirred Tank Reactor
*DAD*: Diode Array Detector
*DP*: Drug Product
*DS*: Drug Substance
*EOS*: Equation of State
*FDA*: The U.S. Food and Drug Administration
*FID*: Flame Ionization Detector
*GC*: Gas Chromatography
*HPLC*: High-Performance Liquid Chromatography
*ICH*: International Council for Harmonization
*IPA*: Isopropyl Alcohol
*LFR*: Laminar Flow Reactor
*MS*: Mass Spectrometry
*NMR*: Nuclear Magnetic Resonance
*NRTL*: Non-Random Two Liquid Model
*PFD*: Process Flow Diagram
*PFR*: Plug Flow Reactor
*RBF*: Round Bottom Flask
*R*_*D*_: Reflux Ratio
*ROH*: Alcohol (e.g., Methanol, Ethanol, Isopropanol)
*RSM*: Regulatory Starting Material
*RTD*: Resistance Temperature Detector
*SM*: Starting Material
*TFF*: Tangential Flow Filter
*USP*: United States Pharmacopeia
*UNIQUAC*: Universal Quasi-chemical Activity Coefficient
*VLE*: Vapor Liquid Equilibrium
*Wt.%*: Weight Percent
