# Supplementary material for: Continuous Distillation for Drug Substance Manufacturing
Source: Pharm Res. 2026 Aug 6;43(7):2301–17. doi: 10.1007/s11095-026-04172-7 (PMC13545118; doi:10.1007/s11095-026-04172-7)
Supplement: Supplementary file 1 [file 11095_2026_4172_MOESM1_ESM.docx]

**Supplementary Information**

**Continuous Distillation for Drug Substance Purification**

**Kimberly E. Penzer, Daniel G. Gregory, Colin D. Thieken, Samantha G. Kohn,
James K. Ferri***

Department of Chemical and Life Science Engineering,

Virginia Commonwealth University, 601 West Main St., Richmond, VA 23284

***** Correspondence: jkferri@vcu.edu; Tel.: (804) 828-7789

**Figure S1.** The process chemistry route employed for the production of albuterol sulfate. Figure adapted with permission from “Development of an Advanced Manufacturing Technology for Continuous Drug Substance Production,” Copyright by Daniel G. Gregory, https://doi.org/10.1021/acs.iecr.5c04185, CC-BY 4.0.

**Figure S2.** A simplified process flow diagram for albuterol sulfate manufacturing within an advanced manufacturing pilot plant (Basis = 1.0 mL/min). Figure adapted with permission from “Development of an Advanced Manufacturing Technology for Continuous Drug Substance Production,” Copyright by Daniel G. Gregory, https://doi.org/10.1021/acs.iecr.5c04185, CC-BY 4.0.

**Figure S3.** Schematic diagram of (**A**) a standard distillation column with central feed and both rectifying (blue) and stripping sections (red), (**B**) diagram of a distillation column with only a rectifying section, and (**C**) diagram of a distillation column for stripping away a lightweight component (e.g., t-butylamine).

**Figure S4.** Scheme depicting the minor dimerized byproducts produced during a side reaction during the upstream S_N_2 amination where k_1_ > k_2_. Copyright by Penzer, DOI: https://doi.org/10.1039/D5RE00465A, CC BY-NC 3.0.

**Figure S5.** Binary vapor liquid equilibrium diagrams as obtained *via* Raoult’s Law in ASPEN+ for TBA/Alcohol mixtures. The top graphs present xy-diagrams for (**A**) TBA in methanol, (**B**) TBA in ethanol, and (**C**) TBA in isopropyl alcohol. The bottom graphs present Txy-diagrams including (**D**) Txy-diagram of TBA in methanol, (**E**) Txy-diagram of TBA in ethanol, and (**F**) Txy-diagram of TBA in isopropyl alcohol. Here x is the liquid mole fraction, and y is the vapor mole fraction.

**Figure S6.** Distillation prototypes including (**A**) a benchtop distillation column, (**B**) a pilot-scale prototype distillation column, (**C**) a continuous distillation column for in-line integration within a continuous manufacturing pilot plant.

| **Description** | **Column Pressure (bar)** | **Feed Temp °C** | **Feed Flow Rate (F) L/min** | **Feed Mass Frac (X_F_)** | **Reflux**  **Ratio** | **Number of Stages (N)** | **Feed Stage** | **Distillate Flow Rate (D)** | **Bottoms Flow Rate (B)** | **Bottoms Concentration (X_B_)** | **Distillate  Concentration (X_D_)** | **Bottoms Temp** | **Distillate Temp** |
| --- | --- | --- | --- | --- | --- | --- | --- | --- | --- | --- | --- | --- | --- |
| Baseline simulation (Exp. 4 conditions) | 1 | 40 | 0.0025 | 0.039 | 1.40 | - | - | 0.00061 | 0.0020 | 0.074 | 0.18 | 81.9 | 74.5 |
| Exp 4 Recreation | 1 | 40 | 0.0025 | 0.039 | 1.40 | 3 | 2 | 0.00061 | 0.0021 | 0.017 | 0.12 | 80.9 | 76.8 |
| Reflux ratio increased by 30% (N = 3) | 1 | 40 | 0.0025 | 0.039 | 1.81 | 3 | 2 | 0.00061 | 0.0021 | 0.017 | 0.11 | 81.0 | 76.7 |
| Reflux ratio increased by 60% (N = 3) | 1 | 40 | 0.0025 | 0.039 | 2.24 | 3 | 2 | 0.00061 | 0.0021 | 0.016 | 0.11 | 81.0 | 76.7 |
| Reflux ratio decreased by 30% (N = 3) | 1 | 40 | 0.0025 | 0.039 | 0.98 | 3 | 2 | 0.00061 | 0.0021 | 0.018 | 0.11 | 81.0 | 76.8 |
| Reflux ratio decreased by 60% (N = 3) | 1 | 40 | 0.0025 | 0.039 | 0.56 | 3 | 2 | 0.00061 | 0.0021 | 0.018 | 0.11 | 81.0 | 76.9 |
| N = 2 stages (RR = 1.40) | 1 | 40 | 0.0025 | 0.039 | 1.40 | 2 | 2 | 0.00061 | 0.0021 | 0.026 | 0.081 | 81.0 | 78.0 |
| N = 4 stages (RR = 1.40) | 1 | 40 | 0.0025 | 0.039 | 1.40 | 4 | 2 | 0.00061 | 0.0021 | 0.011 | 0.13 | 81.2 | 75.9 |
| N = 6 stages (RR = 1.40) | 1 | 40 | 0.0025 | 0.039 | 1.40 | 6 | 2 | 0.00061 | 0.0021 | 0.0048 | 0.15 | 81.5 | 75.0 |
| N = 8 stages (RR = 1.40) | 1 | 40 | 0.0025 | 0.039 | 1.40 | 8 | 2 | 0.00061 | 0.0021 | 0.0048 | 0.15 | 81.5 | 75.0 |
| N = 10 stages (RR = 1.40) | 1 | 40 | 0.0025 | 0.039 | 1.40 | 10 | 2 | 0.00061 | 0.0021 | 0.0020 | 0.16 | 81.6 | 74.6 |

**Table S1.** Aspen sensitivity analysis performed about the validated Experimental Run 4 operating conditions showing the effects of reflux ratio and theoretical stage number while all remaining operating conditions were held constant.

| **Tag** | **Equipment** | **Description** | **Function** | **Critical Process Parameter (CPP)** |
| --- | --- | --- | --- | --- |
| M1303 | Packed Distillation Column | Glass column (½ in. ID, 45 mm OD, 1500 mm packed length), silvered and evacuated; continuously packed with Raschig rings | Vapor–liquid separation | Column temperature profile; column pressure; reflux ratio |
| M1514a | Condenser | Allihn-style overhead condenser | Condensation of overhead vapor | Coolant temperature; condensation efficiency |
| HV-106 | Reflux Divider | Swinging-bucket reflux divider with electromagnet and automated reflux timer | Controls split between reflux and distillate | Reflux ratio; reflux timing |
| M1507 | Reboiler | 250 mL three-neck round-bottom flask with heating mantle (Variac-controlled) | Vapor generation and liquid holdup | Heat duty; bottom temperature |
| — | Vent Valve | Vent located at top of condenser | Pressure relief and control action | Column overhead pressure |
| — | Feed Inlet | Feed introduced at top of column | Continuous feed introduction | Feed flow rate; feed composition; feed temperature |
| M1116 | Bottoms Pump | PTFE-compatible metering pump | Continuous bottoms withdrawal | Bottoms flow rate |
| TT-bottom | Temperature Sensor | Thermocouple at reboiler/column bottom | Monitors reboiler temperature (control point) | Bottom temperature |
| TT-mid | Temperature Sensor | Thermocouple at mid-column | Monitors internal temperature distribution | Mid-column temperature |
| TT-top | Temperature Sensor | Thermocouple near top of packed section | Monitors upper-column temperature | Top-column temperature |
| TT-distillate | Temperature Sensor | Thermocouple in overhead/distillate line | Monitors product thermal condition | Distillate temperature |
| PT-102 | Pressure Sensor | Pressure measurement at column overhead | Monitors column pressure | Column pressure |
| SP-1 | Sampling Port | Bottoms sampling point | Composition analysis of bottoms | Bottoms composition |
| SP-2 | Sampling Port | Distillate sampling point | Composition analysis of distillate | Distillate composition |

**Table S2.** Major equipment, associated functions, and critical process parameters (CPPs) are provided in Table S2 of the Supporting Information.

**Table of Contents Figure:** Continuous Distillation for Drug Substance Manufacturing
